# Spectrum of Fungal Infections in Continuous Ambulatory Peritoneal Dialysis: A 20-Year Retrospective Study From a Tertiary Care Center

**DOI:** 10.7759/cureus.90652

**Published:** 2025-08-21

**Authors:** Sukanya Sudhaharan, Umabala Pamidimukkala, D Sree Bhushan Raju, Nikhi Verma

**Affiliations:** 1 Department of Microbiology, ICMR-Advanced Mycology and Diagnostic Research Centre (AMDRC), Nizam's Institute of Medical Sciences, Hyderabad, IND; 2 Department of Nephrology, Nizam’s Institute of Medical Sciences, Hyderabad, IND

**Keywords:** bipolaris hawaiiensis, molds, paecilomyces variotii, simplicillium obclavatum, yeasts

## Abstract

Introduction

Fungal peritonitis (FP) is a rare but severe complication in patients on continuous ambulatory peritoneal dialysis (CAPD), leading to high mortality. Globally, FP ranges from 1% to 12%, while in India it reaches up to 24%. In the past decades, rare fungi have been identified as the causative agents of FP. The treatment varies depending on the fungi isolated, and it is important to know the species causing infection. The aim of the present study is to analyze the spectrum of fungal pathogens causing CAPD peritonitis. This study highlights a diverse spectrum of fungal pathogens isolated from CAPD patients over 20 years, emphasizing emerging and rare fungi and underscoring the necessity for species-specific antifungal susceptibility data to guide clinical management.

Methods

This was a retrospective observational study of 20 years (January 2004-December 2023) conducted at a tertiary care center in India. The CAPD fluid samples received in the Microbiology Department were inoculated into Sabouraud Dextrose Agar with chloramphenicol and incubated at 30°C and 37°C for 5-7 days. The yeast isolates were identified by the VITEK-2C system (bioMérieux, Marcy-l'Étoile, France). Identification of the molds was done by slide culture of the colony. Antifungal susceptibility testing (AFST) for yeasts was performed as per Clinical and Laboratory Standards Institute (CLSI) guidelines M27. Rare pathogens were identified by sequencing the internal transcribed spacer (ITS) region of the rDNA.

Results

A total of 139 CAPD samples were received from 110 patients. Repeat samples were obtained in 29 patients. Yeast was isolated in 65 (59%) of the samples, whereas mold was isolated in 45 (41%) of the cases. Among the yeasts, *Candida tropicalis (C. tropicalis)* was isolated in 20 (30.7%) cases. Among the molds, *Aspergillus flavus* was isolated in 26 (57.7%) cases.

AFST data could be retrieved for only 33/65 (50.7%) yeast isolates. A total of 27/33 (81.8%) isolates were susceptible to the antifungals tested, and 3/4 (75%)* Trichosporon asahii* (*T. asahii) *isolates were found resistant to amphotericin B. Moreover, 2/20 (10%) *C. tropicalis *isolateswere found resistant to fluconazole and voriconazole. One rare yeast, *Fereydounia khargensis (F. khargensis)*, was resistant to amphotericin B and echinocandins.

Conclusion

*Candida *spp. was the most common pathogen isolated. The spectrum of fungi causing FP and their AFST is important for the appropriate management of patients.

## Introduction

Peritonitis is one of the most frequent complications of peritoneal dialysis (PD) [1,2]. Bacterial peritonitis accounts for 80% of the cases [3], while globally, fungal peritonitis (FP) accounts for 1%-12% of episodes in various studies [4]. In India, the prevalence of FP is 24% [4]. FP is a rare but severe complication leading to high morbidity and mortality [5]. FP should be suspected when there are recurring episodes of bacterial peritonitis and a lack of response to antibiotic treatment. Several fungi have been reported from continuous ambulatory peritoneal dialysis (CAPD)-related peritonitis. *Candida *species are the most common cause of FP, accounting for 70%-90% of the cases [6], but filamentous fungi are now increasingly recognized as a cause of FP. In the last two decades, there has been a steady increase in the spectrum of fungi being reported as opportunistic pathogens in immunocompromised patients [5]. Many soil saprobes and plant pathogens with no obvious pathogenic potential have emerged as etiologic agents, thus posing new diagnostic and therapeutic challenges [7]. FP is less understood and should be diagnosed early, as timely treatment will help prevent the mortality associated with the infections. Identifying the fungus causing infection is essential, as they have varying susceptibility to different antifungals. Also, geographic variations in the prevalence of FP necessitate local studies. The present study aims to analyze the spectrum of fungal pathogens causing CAPD peritonitis. The pathogens were identified by phenotypic/genotypic methods, and their antifungal susceptibility patterns were tested. This study highlights a diverse spectrum of fungal pathogens isolated from CAPD patients over 20 years, emphasizing emerging and rare fungi and underscoring the necessity for species-specific antifungal susceptibility data to guide clinical management. This article was previously presented as a poster at the 2025 Indian Society of Medical Mycologists (ISMM) conference on February 22, 2025.

## Materials and methods

This was a retrospective study with a duration of 20 years (January 2004-December 2023) conducted at a tertiary care center in India. All consecutive CAPD patients with peritonitis in whom fungal pathogens are isolated from the CAPD fluid sample culture are included in the study. Cases of CAPD bacterial peritonitis are excluded from the study.

The data regarding the species causing infections and their antifungal susceptibility pattern were collected from medical and laboratory records. The samples were collected and processed as per the Indian Council of Medical Research (ICMR) mycology standard operating procedure (SOP) [8]. The CAPD fluid samples were inoculated into Sabouraud Dextrose Agar with chloramphenicol. The samples were incubated at 30°C and 37°C for 5-7 days. The yeast isolates were identified by the VITEK-2C system (bioMérieux, Marcy-l'Étoile, France). Identification of the molds was done by slide culture of the colony.

The yeast isolates, which were unidentified by the Vitek2C system, and the mold isolates, which were nonsporulating in culture, were further identified by Sanger sequencing of the internal transcribed spacer (ITS) region of the rDNA. The DNA was isolated using the QIAamp DNA mini kit as per the manufacturer’s protocol (Qiagen, Hilden, Germany). The amplification of DNA was done using ITS 4 and ITS 5 primers to amplify a region in the large subunit (LSU) of ribosomal RNA (rRNA) gene, followed by DNA sequencing and analysis by the Basic Local Alignment Search Tool (BLAST) (National Center for Biotechnology Information (NCBI)).

Due to rapid advancements in fungal taxonomy based on molecular data, some fungal isolates reported in our study have updated nomenclature reflecting current guidelines (e.g., *Meyerozyma guilliermondii,* *Pichia kudriavzevii, Diutina rugosa, *and *Yamadazyma holmii*). Such taxonomic accuracy is critical for precise identification, epidemiological surveillance, and clinical decision-making.

Antifungal susceptibility testing (AFST) for yeasts was done for fluconazole, voriconazole, amphotericin B, caspofungin, and micafungin by VITEK-2C/broth microdilution method as per Clinical and Laboratory Standards Institute (CLSI) guidelines M27 [9]. The major limitation of this study was that AFST was not done for mold isolates, as the methods were not standardized during the study period.

Statistical analysis

Descriptive statistics were used for analysis. Categorical data were described as frequencies with percentages. The data were entered into MS Excel (Microsoft Corporation, Redmond, Washington, United States) and analyzed using IBM SPSS Statistics for Windows, Version 20 (Released 2011; IBM Corp., Armonk, New York, United States).

## Results

A total of 139 CAPD samples were received from 110 patients, and repeat samples were obtained in 29 patients. The predominant age group affected was 51-60 (Table 1). The male-to-female ratio was 4:1.

**Table 1:** Age and sex distribution of the patients (n = 110)

Age group	Male	Female	Total
10-20 years	2	-	2 (1.8%)
21-30 years	6	6	12 (10.9%)
31-40 years	5	2	7 (6.3%)
41-50 years	16	5	21 (19.0%)
51-60 years	34	5	39 (35.4%)
61-70 years	22	3	25 (22.7%)
71-80 years	2	2	4 (3.6%)

Yeast was isolated in 65 (59%) of the samples, whereas mold was isolated in 45 (41%) of the cases. Among yeasts, *Candida tropicalis* (*C. tropicalis*) was isolated in 20 (30.7%) cases, followed by *Candida parapsilosis* (*C. parapsilosis*) (Table 2).

**Table 2:** Yeasts isolated from CAPD samples (n = 65) CAPD:continuous ambulatory peritoneal dialysis *Candida tropicalis* emerged as the predominant yeast isolate with a shift toward non-albicans *Candida*. Rare yeasts such as *Kodamaea ohmeri, Fereydounia khargensis*, and* Rhodotorula glutinis *were isolated. Emerging resistance was observed in *Trichosporon* *asahii* and *Fereydounia*​​​​​​​* khargensis*

Yeast	Number of species	Percentage
Candida tropicalis	20	30.7%
Candida parapsilosis	16	24.6%
Meyerozyma guilliermondii	8	12.3%
Candida albicans	7	10.7%
Trichosporon asahii	4	6.1%
Kodamaea ohmeri	3	4.6%
Trichosporon mucoides	2	3.0%
Fereydounia khargensis	1	1.5%
Diutina rugosa	1	1.5%
Pichia kudriavzevii	1	1.5%
Yamadazyma holmii	1	1.5%
Rhodotorula glutinis	1	1.5%

Among molds, *Aspergillus flavus *was isolated in 26 (57.7%) cases (Table 3).

**Table 3:** Molds isolated from CAPD samples (n = 45) CAPD: continuous ambulatory peritoneal dialysis Note: Rare mold like *Simplicillium obclavatum *was isolated

Moulds	No. of species	Percentage
Aspergillus flavus	26	57.7%
Paecilomyces variotii	5	11.1%
Aspergillus niger	4	8.8%
Aspergillus terreus	2	4.4%
*Acremonium *spp.	2	4.4%
*Fusarium *spp.	2	4.4%
Simplicillium obclavatum	1	2.2%
*Aspergillus flavus *+ *Lichtheimia corymbifera*	1	2.2%
Aspergillus fumigatus	1	2.2%
Bipolaris hawaiiensis	1	2.2%

In all 109 patients, there was isolation of a single yeast/mold. Only one patient showed a mixed etiology of two types of molds (*Aspergillus flavus* and *Lichtheimia corymbifera*).

**Figure 1:**
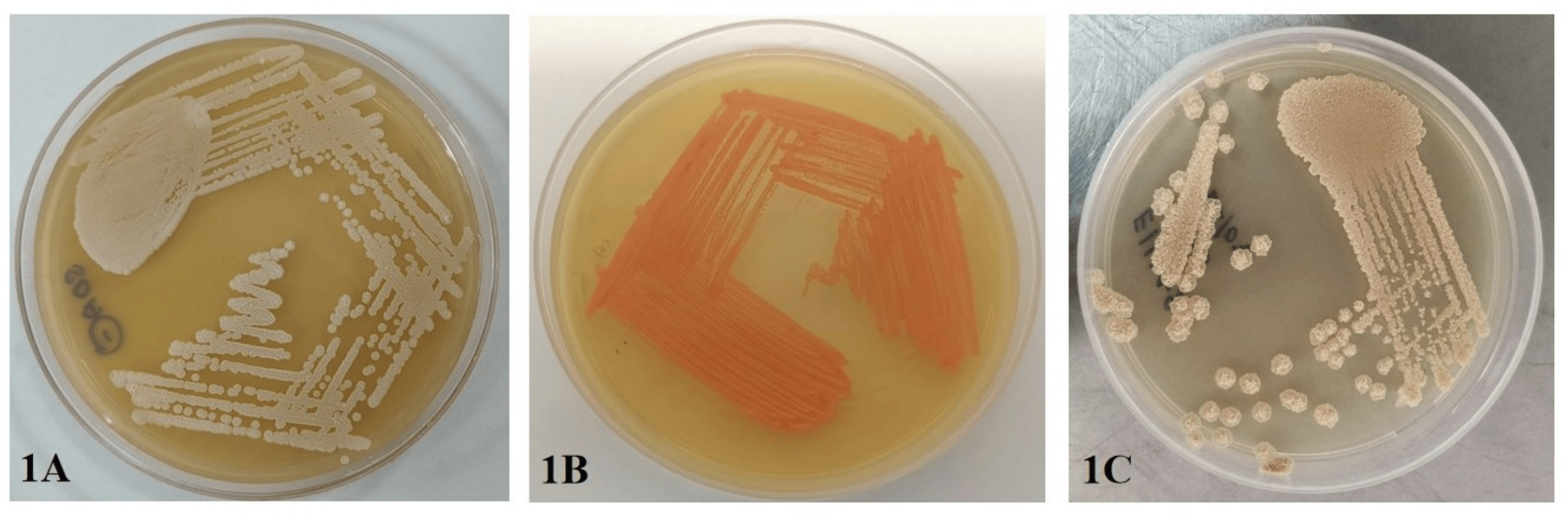
Rare yeasts isolated from CAPD infections CAPD: continuous ambulatory peritoneal dialysis (A) Culture of *Kodamaea ohmeri* on Sabouraud Dextrose Agar showed dry, rough, cream colored colonies. (B) Culture of *Rhodotorula glutinis* on Sabouraud Dextrose Agar showed smooth, glistening, orange color colonies. (C) Culture of *Fereydounia khargensis* on Sabouraud Dextrose Agar showed dry wrinkled colonies

**Figure 2:**
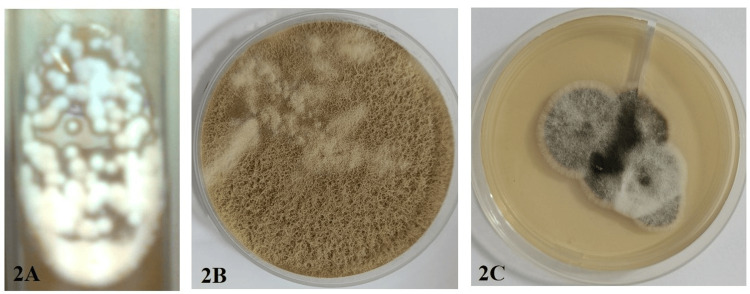
Rare molds isolated from CAPD infections CAPD: continuous ambulatory peritoneal dialysis (A) Culture of *Simplicillium obclavatum* on Sabouraud Dextrose Agar showed white cottony colonies. (B) Culture of *Paecilomyces variotii* on Sabouraud Dextrose Agar showed powdery, yellow-brown colored colonies. (C) Culture of *Bipolaris hawaiiensis* on Sabouraud Dextrose Agar showed hairy black-colored colonies

Rare yeasts *Kodamaea ohmeri (K. ohmeri*)*, Rhodotorula glutinis *(*R. glutinis*), and *Fereydounia khargensis* (*F. khargensis*) were isolated (Figures 1A-1C). Rare molds include *Simplicillium obclavatum (S. obclavatum​​​​​​​*)*, Paecilomyces variotii (P.* *variotii*), and *Bipolaris hawaiiensis* (Figures 2A-2C).

The clinical details were available only for five patients in whom rare fungi were isolated (Table 4).

**Table 4:** Clinical details of five patients with rare fungal pathogens CAPD: continuous ambulatory peritoneal dialysis

S.no	Year	Age/sex	Risk factors	Present complaints	Duration of CAPD	Previous number of episodes of peritonitis	Antibiotic therapy	Causative pathogen	Treatment	Outcome
1	2010	43/M	Hypertension	Fever, abdominal pain, Improper drainage of cloudy dialysate for 5 days	2 years	This is the 1^st^ episode	Nil	Simplicillium obclavatum	Catheter removed. IV Amphotericin B 75 mg for 10 days, and to continue after discharge till a cumulative dose of 2 g is reached	Discharged
2	2011	45/M	Hypertension	Fever, abdominal pain, facial puffiness, and edema for 5 days	2 years	1^st^ episode	Nil	Kodamaea ohmeri	The catheter was removed. Intravenous amphotericin B 50 mg and intravenous fluconazole 200 mg for 10 days, and then oral voriconazole 400 mg twice daily for 14 days	Discharged
3	2012	51/M	Hypertension, right ectopic kidney, absent left kidney	Fever, abdominal pain, vomiting, and turbid effluent for 5 days	2 years	2 episodes	Intravenous imipenem, vancomycin, and amikacin	Paecileomyces variotii	The catheter was removed. Intravenous amphotericin B 50mg for 6 weeks	Discharged
4	2020	30/F	Hypertension	Abdominal pain and cloudy effluents	11 years	5 episodes	Intravenous imipenem, vancomycin, and inj. amikacin intraperitoneal	Paecileomyces variotii	The catheter was removed, and the patient was started on IV voriconazole and IV amphotericin B	Discharged
5	2023	56/f	Autosomal polycystic kidney disease	Nausea, vomiting, abdominal pain, turbid effluent	1 month	1^st^ episode	Intravenous ceftazidime 1.5 g once daily and vancomycin 1 g in 100 ml normal saline over one hour	Fereydounia khargensis	The catheter was removed. Oral fluconazole 200 mg once daily for 5 days, with advice to continue for 2 weeks	Discharged

AFST data could only be retrieved for 33 (52.17%) of the total 65 yeast isolates. Moreover, 27 (81.8%) of the 33 yeast isolates were susceptible to the antifungals tested. Three *Trichosporon asahii *(*T. asahii*) isolates were found resistant to amphotericin B. Two *C. tropicalis* isolates were found to be resistant to fluconazole and voriconazole. One rare yeast, *Fereydounia khargensis*, was found to be resistant to amphotericin B and echinocandins.

## Discussion

FP can be caused by direct entry of the pathogen into the abdominal cavity through the infected catheter exit site or intraluminal contamination of the dialysis tubing [10]. Risk factors for FP include prolonged antibiotic treatment and previous bacterial peritonitis [11]. The peritoneal inflammation caused by bacterial peritonitis increases the susceptibility to fungal invasion, and the antibiotic therapy favors fungal colonization in the skin and digestive system, leading to FP [12].

*Candida albicans* has classically been considered the predominant species. Over the last decade, the number of non-*Candida* albicans species has been growing. Their involvement has become associated with increased mortality, given that some are resistant to the usual antifungals used in treatment [3].

Several fungi have been reported from CAPD-related peritonitis [13,14]. *Candida* species are the most common cause (60-90% of all cases) of fungal peritonitis, of which *C. albicans* is the species most commonly reported [3]. The incidence of CAPD peritonitis caused by non-albicans *Candida* species, especially *C.* ​​​​*parapsilosis, C.* *tropicalis*, and *C.* *glabrata*, is rising [15]. Overall, *C. albicans* and *C. parapsilosis* are the common agents involved, accounting for 70-90% of the cases [12]. FP due to *C. tropicalis* has been rarely reported in literature, ranging from 2.6% to 13% [15,16]. In the present study, *C. tropicalis* was isolated in 20 (30.75%) of the cases, followed by *C. parapsilosis* in 16 (24.6%), which was contrary to the studies in the literature, where *C. tropicalis* was rarely isolated from FP. Also, an increase in antifungal resistance in *Candida *spp. could cause treatment failure and an increase in mortality [1-3]. AFST data could be retrieved only in 33 (52.17%) yeast isolates. Two *C.* *tropicalis* isolates were found to be resistant to fluconazole and voriconazole.

Non-*Candida* fungal species account for less than 10% of cases [17]. The *Trichosporon* genus is a rare cause of FP and causes infection in immunocompromised patients. It colonizes the skin and enters the peritoneal cavity by intraluminal or periluminal surfaces. There are a few reports of CAPD peritonitis caused by *Trichosporon spp*. Due to resistance to echinocandins and fluconazole, it is difficult to treat the infection. Hence, identification of the pathogen is important for proper treatment [18-21]. In the present study, four *T. asahii *and two *T. mucoides* were isolated. Three isolates of *T. asahii* were resistant to amphotericin B and fluconazole.

*Rhodotorula* is a basidiomycete yeast causing opportunistic infection in immunocompromised patients. To date, there are 13 published reports of CAPD FP with *Rhodotorula* spp. *R.* *mucilaginosa* in five cases and *R.* *rubra* in seven cases, and *R. glutinis* in one case [22-24]. In the present study, we isolated *R. glutinis* from a case of FP, which may be the second case of *R.* *glutinis* FP in the literature. As this was a retrospective study, history could not be taken.

*K. ohmeri*, which belongs to the Saccharomycetes family, is usually isolated from the environment and is an emerging pathogen causing severe infections in immunocompromised patients. It causes fungemia in most cases, particularly in neonates [25,26]. There have been nosocomial outbreaks in the pediatric ICU. There are four case reports of *K. ohmeri *CAPD peritonitis [15,27-29]. In the present study, three samples showed growth of *K. ohmeri*. History could be retrieved for one patient who was treated by catheter removal and amphotericin B, fluconazole, and later on with voriconazole.

*F. khargensis* is an emerging new yeast in the order Urocystales and was isolated from plant remnants in Iran. The yeast can be identified by molecular methods. To date, there have been two cases reported in the literature. It is resistant to amphotericin B and echinocandins [30,31], as in our study.

Several filamentous fungi, less frequently, cause FP. However, both hyaline and melanized fungi, such as *Aspergillus* species, Mucormycetes, *Fusarium* spp., *Paecilomyces* spp., and *Curvularia* spp., are being recognized as a cause of mold peritonitis [1,3]. 

*Aspergillus* peritonitis is rare, accounting for 2-5% of FP, but mortality is 15-50% higher compared to *Candida* spp. Several species of *Aspergillus* have been reported to cause FP. In a review of 55 cases of aspergillosis, *Aspergillus fumigatus* was isolated in 17 of 55 cases, followed by *Aspergillus niger* [12]. Besides other risk factors, long-term peritoneal dialysis of more than 36 months can lead to *Aspergillus *peritonitis [32]. In the present study, *Aspergillus* spp. were isolated from 34 samples. *Aspergillus flavus* was the most commonly isolated species, likely due to its ability to survive in the hot and arid climates typical of tropical countries, such as India.

*Pacileomyces *spp. is an environmental pathogen that is a rare but recognized cause of CAPD peritonitis. There are a few reports of peritonitis caused by *Pacileomyces *spp. [33,34]. In the present study, *P. variotii *was isolated in five patients; however, history was retrieved for two patients. The patients had previous episodes of bacterial peritonitis and were successfully treated with amphotericin B and voriconazole.

*S. obclavatum* is a plant pathogen that is present in the environment and has not been reported to cause human infection [35]. In the present study, we isolated the pathogen from a 43-year-old male patient, hypertensive with end-stage renal disease on CAPD for two years. The organism was isolated twice from a culture of CAPD fluid, sent one month apart. The culture was nonsporulating and sent to the National Culture Collection of Pathogenic Fungi (NCCPF). At NCCPF, molecular identification was done by sequencing the ITS region of the rDNA, and it was identified as *S. obclavatum.* The catheter was removed, and the patient was started on amphotericin B and discharged in a stable condition. To the best of our knowledge and literature search, this is the first case report of CAPD peritonitis caused by *S. obclavatum.*

*Acremonium* spp. are found in nature and cause opportunistic infections. The organism is usually resistant to antifungal therapy. The infection usually occurs through environmental contamination. There were seven cases of *Acremonium* peritonitis reported till 2023 [36]. In the present study, two cases of *Acremonium* spp. were isolated. Speciation could not be done on isolates. Further molecular identification would have classified these isolates differently.

*Fusarium* is present in the soil and organic debris. It causes a variety of infections, but there are few reports of *Fusraium* peritonitis. It causes infection in patients on long-standing dialysis for more than two years. Tissue breakdown is the common site of entry of *Fusarium* [37-39]. In the present study, two isolates of* Fusarium* were isolated. Speciation was not for the isolates.

*Bipolaris* is an uncommon cause of FP. There were two reports of *Bipolaris* peritonitis in the literature that were successfully treated by removal of the catheter and antifungal therapy [40,41]. In the present study, *Bipolaris hawaiiensis* was isolated from one sample.

Knowledge of the pathogen causing infection and identification of the pathogens by culture/molecular methods is important, as they have varied susceptibility to different antifungals. Antifungal susceptibility testing should be done, which would help in targeted treatment and thereby proper management of the patients. In the present study, AFST was done only for yeast isolates.

Treatment guidelines recommend removing the catheter followed by appropriate antifungal therapy for at least two weeks after catheter removal [42]. A catheter left in situ after diagnosing fungal peritonitis is associated with a worse prognosis, with a greater rate of technical failure and an increase in mortality, as reported in several series [1-3,10].

Limitations of the study

The major limitation of this study included the following: As this was a retrospective study, clinical history, treatment, or outcomes of patients could not be collected for all patients except for five isolates. Hence, data and statistical analyses such as chi-square tests or odds ratios, on outcomes like mortality, resistance, or infection recurrence, could not be calculated. While all described patients were discharged, the retrospective nature and lack of follow-up data limit our conclusions regarding long-term prognosis, clinical success, or recurrence rates. AFST was not done for mold isolates, as the methods were not standardized during the study period. Prospective multicentric studies with fungal identification and AFST, along with complete clinical details of the patient, will provide better knowledge of FP in our geographic location.

## Conclusions

The present study highlights the spectrum of fungi causing CAPD fungal peritonitis. *Candida* spp. was the most common pathogen isolated. The majority of the yeast isolates were found to be susceptible to antifungals. Rare yeast and molds, which were environmental pathogens, were also isolated. Care should be taken in the maintenance of the catheter as the pathogens gain entry through the site of insertion. The spectrum of fungi causing FP and their antifungal susceptibility testing is important for the appropriate management of patients. Our findings reinforce the necessity for routine antifungal susceptibility testing, early catheter removal upon fungal identification, and heightened surveillance for rare pathogens to improve patient outcomes in CAPD fungal peritonitis.
